# From cup to dish: how to make and use endometrial organoid and stromal cultures derived from menstrual fluid

**DOI:** 10.3389/fendo.2023.1220622

**Published:** 2023-09-21

**Authors:** Sylvia C. Hewitt, Mackenzie J. Dickson, Nicole Edwards, Kathleen Hampton, Stavros Garantziotis, Francesco J. DeMayo

**Affiliations:** ^1^ Reproductive and Developmental Biology Laboratory, National Institute of Environmental Health Sciences (NIEHS), Durham, NC, United States; ^2^ Clinical Research Unit, National Institute of Environmental Health Sciences (NIEHS), Durham, NC, United States

**Keywords:** menstrual fluid, endometrium, organoid culture, stroma cell culture, method

## Abstract

Diseases impacting the female reproductive tract pose a critical health concern. The establishment of *in vitro* models to study primary endometrial cells is crucial to understanding the mechanisms that contribute to normal endometrial function and the origins of diseases. Established protocols for endometrial stromal cell culture have been in use for decades but recent advances in endometrial organoid culture have paved the way to allowing study of the roles of both epithelial and stromal endometrial cells *in vitro*. Due to inter-individual variability, primary cell cultures must be established from numerous persons. Generally, endometrial epithelial and stromal cells can be isolated from an endometrial biopsy, however, this is collected in a clinical setting by an invasive transcervical procedure. Our goal was to develop a non-invasive method for the isolation of paired endometrial epithelial organoids and stromal cells from menstrual fluid collected from individual women, based on recent reports describing the isolation of endometrial epithelial organoids or endometrial stromal cells from menstrual fluid. Participants recruited by the NIEHS Clinical Research Unit were provided with a menstrual cup and instructed to collect on the heaviest day of their menstrual period. Endometrial tissue fragments in the menstrual fluid samples were washed to remove blood, minced, and digested with proteinases. Following digestion, the solution was strained to separate epithelial fragments from stromal cells. Epithelial fragments were washed, resuspended in Matrigel, and plated for organoid formation. Stromal cells were separated from residual red blood cells using a Ficoll gradient and then plated in a flask. Once established, estrogen responsiveness of endometrial epithelial organoids was assessed and the decidual response of stromal cells was evaluated. Following treatments, qPCR was performed on organoids for genes induced by estradiol and on stromal cells for genes induced by decidualization. In this manner, the relative responsiveness of paired organoid and stroma cell cultures isolated from each woman could be assessed. In conclusion, we can isolate both epithelial and stromal cells from a single menstrual fluid sample, allowing us to establish organoids and cells in a paired manner. This protocol can greatly enhance our knowledge of the role of epithelial and stromal cells alone and in coordination.

## 1 Introduction

Diseases impacting the endometrium pose a critical health concern to women as well as to their children. Establishing an optimally receptive endometrium lays the groundwork for a healthy pregnancy, which can greatly impact the life-long health of an individual (1, 2). Uterine development can be disrupted by exposures to substances in the environment or by disrupting normal endocrine signals including estrogen (3). Problems with implantation or placentation can lead to complications such as hemorrhage or pre-eclampsia (2, 4) and the necessity of undesired interventions including bedrest or pharmacological treatments, which might inadvertently impact the developing fetus. *In vitro* models that recapitulate aspects of endometrial cell biology are crucial for understanding and testing the mechanisms regulating endometrial function and disease. Protocols for endometrial stromal cell culture are well established and can effectively model hormone-dependent processes *in vitro* (5). Processes intrinsic to epithelial cells of the endometrium, which are essential for embryo attachment and implantation, along with the secretion of key receptivity factors to support the pregnancy until the placental blood supply is fully developed (2, 6), are not captured by stromal cultures. Until recently, growing primary endometrial epithelial cells in culture has not been very successful. Advances in endometrial organoid culture have paved the way to allowing scientists to study the roles of both epithelial and stromal cells *in vitro* (7, 8). Due to variability between donors, primary cell cultures must be established from numerous individuals. Generally, endometrial epithelial and stromal cells can be isolated from an endometrial biopsy, however, this is collected in a clinical setting by an invasive transcervical procedure. Our goal was to develop a non-invasive method for the isolation of paired endometrial epithelial organoids and stromal cells from menstrual fluid collected in a reusable menstrual cup (9) from individual women, based on recent reports describing the isolation of endometrial epithelial organoids or endometrial stromal cells from menstrual fluid (10–12).

## 2 Reagents, materials, and equipment

• Instructions to participants
• Menstrual cups such as Diva cup brand and packaging with instructions
• Specimen container for sample (such as Coviden 2210SA)
• Water Bath
• Cell culture incubator (37°C with 5% CO2)
• Biological Safety Cabinet
• Refrigerated Centrifuge with carriers for 15 and 50 ml conical tubes, capable of centrifugation at 600 RCF, with the ability to turn off the brake.
• Pipettes and tips
• Sterile tubes: 50 mL, 15 mL, and 1.5 mL
• Cell counter or Hemocytometer
• PBS-Calcium Magnesium Free
• Penicillin-Streptomycin (p/s;Sigma, P0781)
• Fungizone (Amphotericin B; Sigma, A2942)
• 40 µm cell strainers (Falcon, 352340)
• 100 µm cell strainers (Falcon, 352360)
• Ficoll-Paque Plus (Cytiva, 17144002)
• RPMI Medium 1640 (Gibco, 11835-030)
• Dispase II (Sigma, D4693)
• Collagenase V (Sigma, C-9263)
• Fetal Bovine Serum (Thermo Fisher 10082147)
• Matrigel (Corning, 536231)
• 12-well tissue culture dishes
• 100 mm tissue culture plates
• T25 flasks
• Trypsin-EDTA (0.25%) (Gibco, 25200-056)
• DMSO (Cell culture grade)
• Trizol Reagent (Thermo Fisher 15596018)
• Chloroform
• Glycogen (20 mg/ml, Millipore Sigma 10901393001)

### 2.1 PBS+p/s/f or RPMI+p/s/f or advanced DMEM+p/s/f

• Per 500 mL PBS or RPMI or Advanced DMEM/F12:
• 5 mL pen/strep (1%)
• 5 mL fungizone (1%)

### 2.2 Tissue digestion solution

• Add the ingredients below then filter with a syringe filter (0.2 µm). Store on ice until digestion.
• 20 mL RPMI+ p/s/f
• 500 µl 100 mg/ml Dispase II solution (final concentration 1.25 U/ml Dispase II)
• 800 µl 10 mg/ml Collagenase V (final concentration 0.4mg/ml collagenase V)

### 2.3 Stop solution

• Add the ingredients below then filter with a syringe filter (0.2 µm)
• 90 mL RPMI+p/s/f
• 10 mL FBS (10%)

### 2.4 Organoid expansion media

See **Table 1**

**Table 1:** Human Endometrial Epithelial Organoid Medium.

Base Medium	Total volume of Base Medium: 500ml				
Product	Company	Catalog #	Stock concentration	Final concentration in 500ml Advanced DMEM/F12	Volume in 500ml Advanced DMEM/F12
Advanced DMEM/F12 1X	Gibco	12634-010 (500ml)	1x	1X	500ml
B27 Supplement	Gibco	12587-010 (10ml)	50X	1X	10ml
Insulin-transferrin-selenium	Gibco	41400-045 (10ml)	100X	1X	5.0ml
Primocin	InvivoGen	ant-pm (1ml)	50mg/ml	100µg/ml	1ml
Glutamax	Gibco	35050-061	100X	1X	5.0ml
Expansion Medium (ExM)	Total volume of ExM: 50ml				
Product	Company	Catalog #	Stock concentration	Final concentration in 50ml Base Medium	Volume in 50ml Base Medium
Base Medium (as above)					50ml
N2	Gibco	17502-048	100X	1X	500µl
N-acetyl-L-cysteine	Sigma	A9165 (MW: 163.19g/mol)	Make a 625mM solution in sterile dH2O (2.04g in 20ml MilliQ H2O)	1.25mM	100µl
A83-01	Tocris Biosciences	2939	Make a 500 µM solution in DMSO (1.05mg in 5ml DMSO)	500nM	50µl
HGF	Peptrotech	100-39	50µg/ml (reconstitute in sterile dH2O)	50ng/ml	50µl
EGF	Peptrotech	AF-100-15	500µg/ml (reconstitute in sterile dH2O)	50ng/ml	5µl
FGF-10	Peptrotech	100-26	100µg/ml (reconstitute in sterile dH2O)	100ng/ml	50µl
R-spondin	Peptrotech	120-38	500µg/ml (reconstitute in sterile dH2O)	500ng/ml	50µl
Noggin	Peptrotech	120-10C	100µg/ml (reconstitute in sterile dH2O)	100ng/ml	50µl
Nicotinamide	Sigma	N0636	10mM then dilute 1:1000 to make 0.01mM solution (sterile dH2O) 122.12 g/mol	10nM	50µl of the 0.01mM stock
Y27632	Tocris Biosciences	1254	Dissolve 1 mg in 312 ul H2O	10 uM	50µl
Fungizone - Amphotericin B	Sigma	A2942	100x	1%	500µl
Base Medium-B27	Total volume of Base Medium: 500ml				
Product	Company	Catalog #	Stock concentration	Final concentration in 500ml Advanced DMEM/F12	Volume in 500ml Advanced DMEM/F12
Advanced DMEM/F12 1X	Gibco	12634-010 (500ml)	1x	1X	500ml
Insulin-transferrin-selenium	Gibco	41400-045 (10ml)	100X	1X	5.0ml
Primocin	InvivoGen	ant-pm (1ml)	50mg/ml	100µg/ml	1ml
Glutamax	Gibco	35050-061	100X	1X	5.0ml
Hormone Treatment Medium (ExM-N2)*	Total volume of ExM: 50ml				
Product	Company	Catalog #	Stock concentration	Final concentration in 50ml Base Medium	Volume in 50ml Base Medium
Base Medium-B27 (as above)					50ml
N-acetyl-L-cysteine	Sigma	A9165 (MW: 163.19g/mol)	Make a 625mM solution in sterile dH2O (2.04g in 20ml MilliQ H2O)	1.25mM	100µl
A83-01	Tocris Biosciences	2939	Make a 500 µM solution in DMSO (1.05mg in 5ml DMSO)	500nM	50µl
HGF	Peptrotech	100-39	50µg/ml (reconstitute in sterile dH2O)	50ng/ml	50µl
EGF	Peptrotech	AF-100-15	500µg/ml (reconstitute in sterile dH2O)	50ng/ml	5µl
FGF-10	Peptrotech	100-26	100µg/ml (reconstitute in sterile dH2O)	100ng/ml	50µl
R-spondin	Peptrotech	120-38	500µg/ml (reconstitute in sterile dH2O)	500ng/ml	50µl
Noggin	Peptrotech	120-10C	100µg/ml (reconstitute in sterile dH2O)	100ng/ml	50µl
Nicotinamide	Sigma	N0636	10mM then dilute 1:1000 to make 0.01mM solution (sterile dH2O) 122.12 g/mol	10nM	50µl of the 0.01mM stock
Y27632	Tocris Biosciences	1254	Dissolve 1 mg in 312 ul H2O	10 uM	50µl
Fungizone - Amphotericin B	Sigma	A2942	100x	1%	500µl

*N2 and B27 are omitted from hormone treatments as they contain some progesterone.

### 2.5 Stromal cell (HESC) culture media

See **Table 2**

**Table 2:** Human Endometrial Stromal Cell (HESC) Medium.

Culture Medium	Total volume of Culture Medium: 500ml			
Product	Company	Catalog #	Final concentration in 500ml DMEM/F12	Volume in 500ml DMEM/F12
DMEM/F12 1X	Gibco	11330-057 (500ml)	1X	500ml
Fetal Bovine Serum (FBS)	Gibco/Thermo Fisher	10082147	10%	50ml
Penicillin-Streptomycin	Sigma	P0781	1%	5 mL
Fungizone - Amphotericin B	Sigma	A2942	1%	5 mL

#### 2.5.1 HESC decidual media

See **Table 3**

**Table 3:** Human Endometrial Stromal Cell (HESC) Decidualization Medium.

Culture Medium	Total volume of Culture Medium: 500ml			
Product	Company	Catalog #	Final concentration in 500ml DMEM/F12	Volume in 500ml DMEM/F12
OptiMem	Gibco	31985070	1X	500ml
Charcoal Stripped Fetal Bovine Serum (c	Gibco/Thermo Fisher	12676029	2%	10ml
Penicillin-Streptomycin	Sigma	P0781	1%	5 mL

1000x estradiol (E2) solution: 10 µM E2 (Steraloids, Inc E0950-000) dissolved in 100% ethanol.
1000x medroxyprogesterone acetate (MPA) solution: 1 mM MPA (Sigma M1629) dissolved in 100% ethanol.
1000x cyclic AMP (cAMP): 100 mM cAMP (Sigma D0627) dissolved in autoclaved water.
HESC decidual media + V: add 2 µl 100% ethanol and 1 µl sterile water per ml
HESC decidual media + EPC: add 1 µl 1000x E2, l µl 1000x MPA and 1 µl 1000x cAMP per ml HESC Decidual Media.

#### 2.5.2 Organoid hormone treatment media (ExM-N2)

See **Table 1**

ExM-N2+V: add 1 µl 100% ethanol per 1 ml ExM-N2.

ExM-N2+E2: add 1 µl 1000x E2 per 1 ml ExM-N2.

## 3 Methods

1. Under a NIEHS Institutional Review Board-approved protocol (000152) all donors must provide informed consent prior to specimen collection. Provide the participant with a menstrual cup, instructions, a specimen container, an ice pack, a biohazard bag, and a padded envelope. The participant is instructed to collect on their heaviest flow day for 6-12 hours and store it in a refrigerator.
2. Arrange drop off or courier transport of the collected sample in a specimen cup packaged in a biohazard bag inside a padded envelope with a frozen ice pack when it is available, ideally within 24-48 hours of collection. It is important to stress to the participants the importance of storing the sample at 4°C, as the integrity of the sample will diminish.
Note: the remaining steps where the sample is exposed (open container) are performed in a biological safety cabinet
3. Transfer menstrual fluid from the specimen container into a 50 mL tube. Note the approximate volume of menstrual fluid. Rinse the container with cold PBS+p/s/f and transfer to a 50 mL tube.
3a. If the original sample volume is greater than 15 mL, split the sample so that each 50 mL tube contains material from 5-7 mL of sample.
4. Fill all tubes to 50 mL with cold PBS+p/s/f. Invert to mix.
5. Centrifuge sample at 600 RCF for 5-10 minutes at 4°C. Carefully remove the supernatant with a pipette, and avoid disrupting the pellet. Discard the supernatant.
6. Resuspend the pellet to 50 mL with cold PBS+p/s/f and centrifuge at 600 RCF for 5-10 minutes at 4°C, remove and discard the supernatant. Repeat resuspending in cold PBS+p/s/f and centrifuging until the supernatant is clear and colorless. Normally, this takes at least 4x.
7. Add cold PBS+p/s/f to pellets; if the sample was split into multiple tubes in step 3a, re-combine the material into one tube. Pass through a 100 µm cell strainer placed on top of a 50 mL tube. Avoid large fragments, clots, and mucus at first to facilitate flow through the strainer, but then pass all material through the strainer. If the strainer clogs, pass the remaining material through a new 100 µm cell strainer. Wash the strainer with more cold PBS+p/s/f. Wash until fragments remain in the strainer and most blood is rinsed off.
8. Invert the cell strainer over a 100 mm dish and backwash tissue fragments into a dish with cold PBS+p/s/f using a transfer pipette until all tissue is in the dish.
9. Chop tissue with scissors and forceps into small pieces (2-3 mm). Rinse with cold PBS+p/s/f to remove blood if needed.
10. Using forceps, transfer the tissue fragments to a 50 mL tube containing 20 mL tissue digestion solution.
11. Incubate in a water bath at 37°C for 20 minutes, inverting every 5 minutes to digest the tissue.
12. To stop the digestion, add 20 mL of a cold stop solution. Mix by pipetting up and down 4+ times with a 25 mL pipette, and pass through a clean 100 µm cell strainer set on top of a clean 50 mL tube.

Cell strainer contains epithelial fragments (continue with Step 13).

Filtrate (in the 50 mL tube) contains stromal cells (go to Step 18).

### 3.1 Epithelial fragments/organoids

13. Invert the cell strainer over a 100 mm dish, and backwash retained epithelial fragments from the cell strainer with a cold stop solution into the dish using a transfer pipette until all fragments are in the dish.
14. Transfer the fragments from the 100 mm dish to a 15 mL tube and centrifuge for 10 minutes at 400 RCF at 4°C.
15. Resuspend the pellet in 150-200 µl ice-cold Matrigel.
16. Plate three to four 25-40 µl drops of Matrigel-cell suspension per well of a pre-warmed 12-well culture dish and incubate at 37°C for 15 minutes.
17. Add 750 µl Organoid Expansion Media.

### 3.2 Stromal cells

18. Pass 100 µm filtrate containing stromal cells through a 40 µm cell strainer set on top of a clean 50 mL tube. Pellet stromal cells by centrifugation at 400 RCF for 10 minutes at 4°C.
19. Remove the supernatant and resuspend the stromal cell pellet in 4 mL of HESC culture medium (should use phenol red-containing media as it helps to visualize Ficoll interface) and layer on 4 mL Ficoll in a 15 mL tube.
20. Centrifuge the sample overlaid on Ficoll for 20 minutes at 400 RCF with the brake off.
21. Collect cells from the interface, add a warm HESC culture medium, and centrifuge at 400 RCF for 10 minutes.
22. Resuspend the stromal cell pellet in 3 mL warm HESC culture medium and place cell suspension in a T25 flask.
23. Incubate at 37°C for 20 minutes and then transfer medium from this first flask into a second flask. Replace the medium in the first flask. Most stromal cells should be in the first flask and the second flask should contain stromal cells that did not adhere to the first flask as well as contaminating epithelial, blood, and immune cells.
24. After 60+ minutes, only stromal cells will have adhered to both flasks. Wash both flasks vigorously with room temperature PBS+p/s/f to remove other cell types and add 5 ml fresh warm HESC culture medium.
25. For both culture types (organoids and stromal cells), change media every 2-3 days.

### 3.3 Passaging

26. Passage stromal cells using a standard trypsinization protocol.
27. Passage organoids by dislodging Matrigel droplets with a p1000 tip and pipetting up and down in culture media to disrupt the Matrigel and organoid structure. Transfer to a 15 mL tube.
28. Rinse the wells with 1 mL cold Advanced DMEM+p/s/f, add to 15 mL tube from Step 27, and centrifuge at 400 RCF for 10 minutes at 4°C.
29. Remove supernatant, pipette 1 mL cold Advanced DMEM onto pellet, pipet up and down to dissolve Matrigel. Add 1 mL more of cold Advanced DMEM and centrifuge at 400 RCF for 10 minutes at 4°C.
30. Remove the supernatant and resuspend the pellet in ice-cold Matrigel. Pipette to mix. Plate three to four 25-40 µl drops of Matrigel-cell suspension per well of a pre-warmed 12-well culture dish.
31. Incubate at 37°C for 15 minutes, then add 750 µl Organoid Expansion Media.

### 3.4 Cryopreservation and Cryorecovery

#### 3.4.1 HESC

32. For cryopreservation, stromal cells (10^6^ cells per vial) should be frozen in a HESC culture medium containing 10% DMSO. Freeze in Mr. Frosty, or equivalent, for 24 hours in a -80°C freezer, and store long term in a liquid nitrogen freezer.
33. For cryo recovery of frozen HESC, thaw the vial in a 37°C water bath, add to 9 ml HESC culture medium, centrifuge 400 RCF for 10 minutes to recover cells, resuspend HESC in 10 ml pre-warmed HESC culture medium, and plate in T75 flask or 100 mm tissue culture dish. Cells recover well, on par with the recovery of biopsy-derived HESC.

#### 3.4.2 Organoids

33. For cryopreservation of organoids, the amount of harvested organoid fragments that would be plated in 3-4 Matrigel drops should be frozen per vial in FBS containing 10% DMSO. Freeze in Mr. Frosty, or equivalent, for 24 hours in a -80°C freezer, and store long term in a liquid nitrogen freezer.
34. For cryo recovery of frozen organoids, thaw by pipetting 500 µl pre-warmed Advanced DMEM into cryovial and transferring thawed contents into a 15 ml tube. Repeat until all vial contents are thawed. Centrifuge at 400 RCF for 10 minutes at 4°C.
35. Remove supernatant, pipette 1 mL cold Advanced DMEM onto pellet, pipet up and down, and centrifuge at 400 RCF for 10 minutes at 4°C.
36. Remove the supernatant and resuspend the pellet in ice-cold Matrigel. Pipette to mix. Plate three to four 25-40 µl drops of Matrigel-cell suspension per well of a pre-warmed 12-well culture dish. Incubate at 37°C for 15 minutes, then add 750 µl Organoid Expansion Media. Organoids form and grow slowly after thawing but recover well.

### 3.5 Testing hormone responses of cultured cells

#### 3.5.1 HESC

37. Plate six wells of 100,000 to 150,000 cells per well in a 6-well dish in a HESC culture medium.
38. Incubate for 24 hours.
39. Aspirate medium, wash 1x with PBS.
40. To three wells add HESC Decidual Media with EPC added and to three wells add HESC Decidual Media with V added.
41. after 48 hours change the media with fresh HESC Decidual Media with EPC or V.
42. After 24 hours (a total of 72 hours after adding EPC or V), take photos of the cells.
43. To isolate RNA, aspirate media and wash 1x with PBS.
44. Add 1 ml Trizol Reagent to each well.
45. Scrape each well and transfer the solution to 1.5 microcentrifuge tubes and vortex to mix. Let samples sit for 5 minutes.
46. Add 200 µl of chloroform to each tube, vortex for 30 seconds, let sit for 2-3 minutes, repeat vertexing.
47. Centrifuge in refrigerated microfuge, max RPM at 4°C for 10 minutes.
48. Carefully remove the aqueous (top) phase and transfer each sample to a clean 1.5 ml tube.
49. Add 1 µl Glycogen to each sample and mix.
50. Add 500 µl 2-Propanol to each sample. Vortex to mix.
51. Incubate samples at -20°C for 30 minutes.
52. Spin in refrigerated microfuge, max RPM, 4°C, for 10 minutes, then carefully remove supernatant; do not disturb RNA pellet.
53. Rinse pellet with cold 75% EtOH.
54. Leave the tube open to dry the pellet well.
55. Add 50 μl DEPC H2O and allow the RNA pellet to dissolve.
56. Measure RNA concentration. Make cDNA and utilize RT-PCR to measure gene expression.

#### 3.5.2 Organoids

57. Plate six wells with three droplets per well in a 12-well dish.
58. Culture in ExM for 4 days, changing ExM after 2 days, to allow organoids to form.
59. Change media to three wells of ExM-N2+V and three wells of ExM-N2+E2.
60. Change media every 2-3 days.
61. On the 5^th^ day of hormone treatment, change the media and harvest the organoids 6 hours later.
62. For each well, mix media and droplets with P1000, transfer to 1.5 ml microcentrifuge tube.
63. Centrifuge at 600 RCF for 10 minutes at 4°C.
64. Remove media+Matrigel.
65. Resuspend in 500 µl cold Base Media.
66. Centrifuge at 600 RCF for 10 minutes at 4°C.
67. Remove Matrigel and supernatant.
68. Resuspend each pellet in 200 µl of Trizol Regent.
69. Homogenize/vortex to lyse the cells.
70. Add 300 µl additional Trizol and mix.
71. Follow steps 46-56 for HESC above.

## 4 (Anticipated) Results

**Figure 1:**
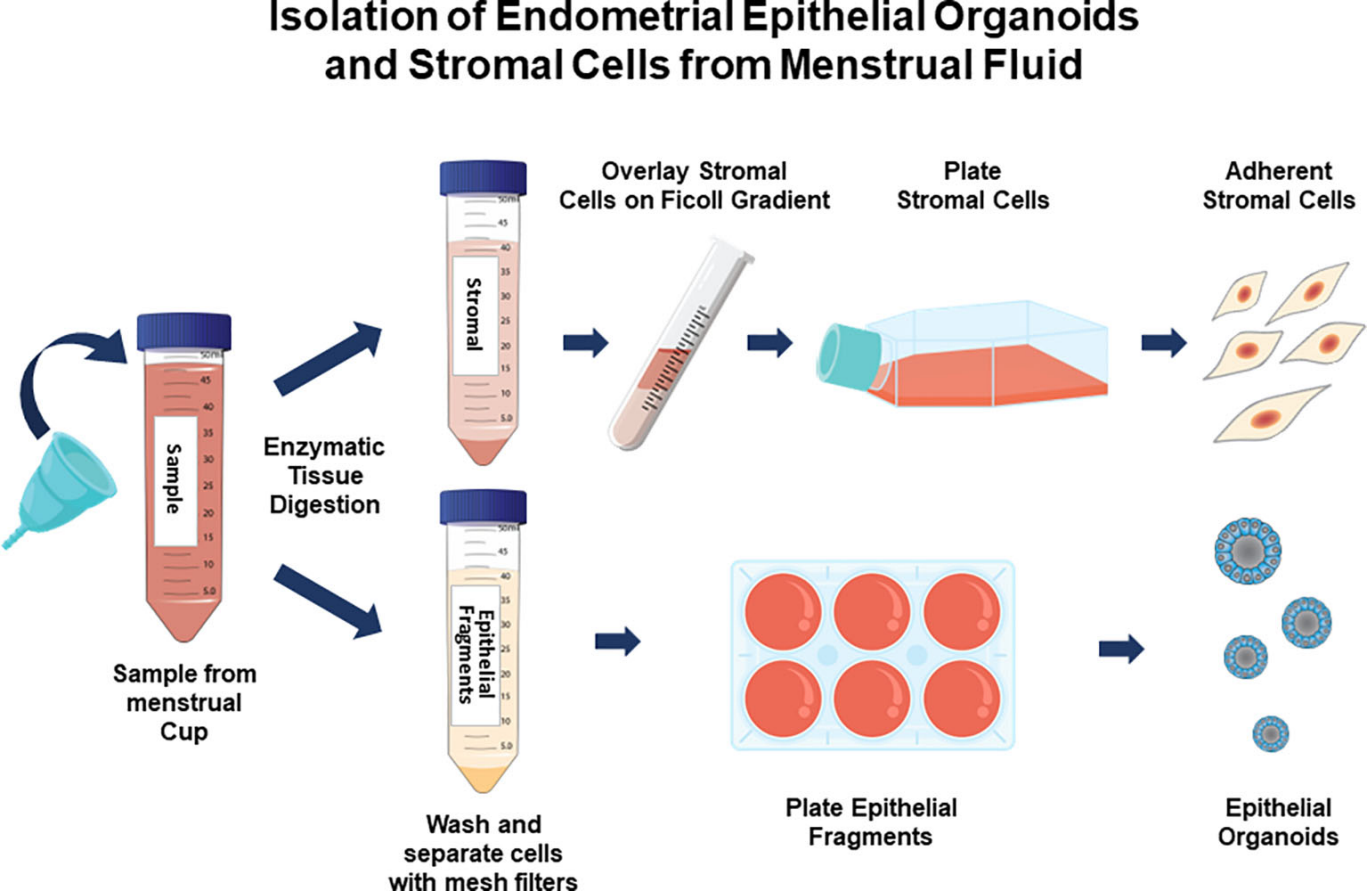
Isolation and culture of endometrial stromal cells and epithelial organoids from menstrual fluid samples. Participants collect menstrual fluid in a menstrual cup and provide it to the clinical center. Endometrial tissue fragments are digested with proteinases and stroma cells are separated from epithelial cell fragments. Stroma cells are separated from blood cells on a Ficoll gradient and cultured as adherent monolayers. Epithelial cells are cultured in Matrigel, forming organoids.

**Figure 2:**
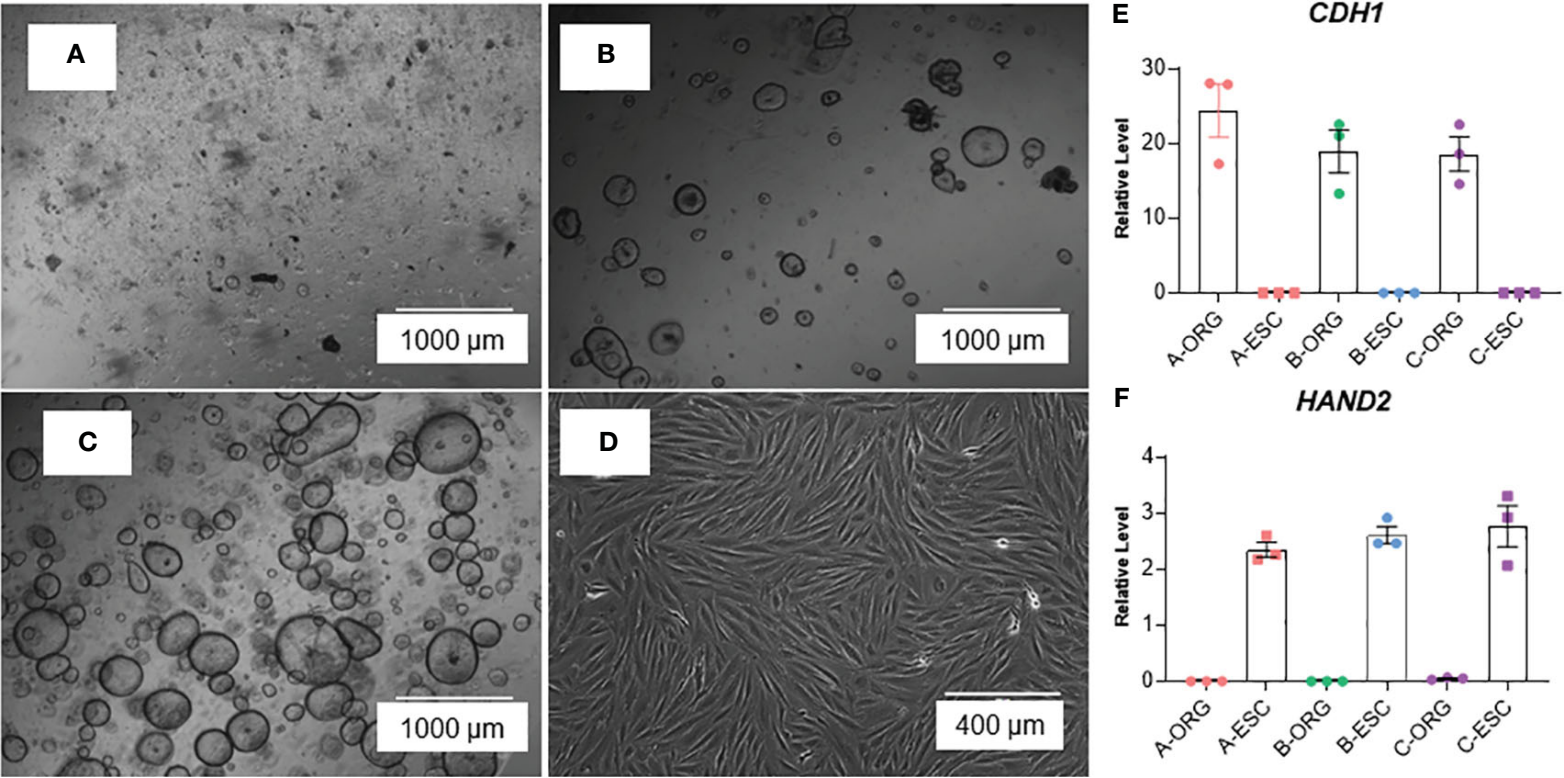
Images of organoids forming **(A)** passage 0, 1 day after isolation/plating, **(B)** passage 1, 1 day after plating, and **(C)** passage 2, 2 days after plating) and **(D)** stromal cells at confluency. **(E, F)** RT-PCR of RNA isolated from organoid (ORG) or stroma cell (ESC) cultures; E: epithelial cell marker, E-Caherin (*CDH1*) and F: stroma cell marker Heart And Neural Crest Derivatives Expressed 2 (*HAND2*). Samples were isolated from cultures derived from three different participants **(A–C)**. Primer sequences are listed in **Table 4**.

**Figure 3:**
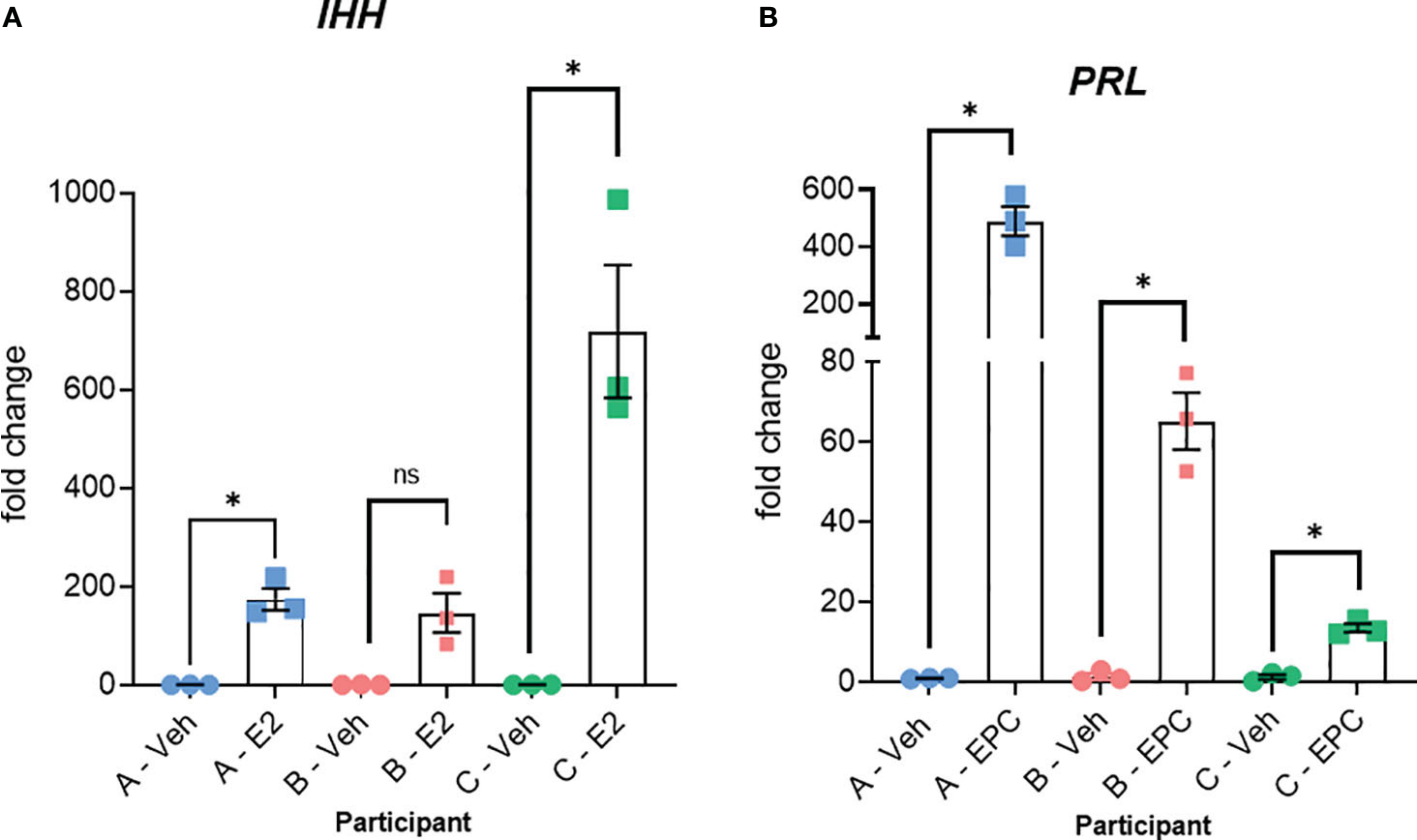
Relative responses of epithelial organoid and stromal cell cultures from three different individuals. **(A)** RT-PCR for *IHH* of RNA isolated from organoid cultures treated with vehicle (Veh) or estrogen (E2) for 6 days + 6 hours as described previously (13). Organoids were isolated from three different individuals (participants A, B, and C). **(B)** RT-PCR for the decidual cell marker *PRL* of RNA isolated from stromal cultures treated with vehicle (Veh) or 10 nM estradiol, 1 µM medroxyprogesterone acetate, and 100 µM cAMP (EPC) for 3 days to induce decidualization as described previously (14). Stromal cells isolated from the same three participants (A, B, and C) were used. Primer sequences are listed in **Table 4**. Significance was analyzed using paired t-tests in GraphPad Prism with *, p<0.05.

The method is summarized in the graphic in **Figure 1**. Images of organoids and stromal cells are shown in **Figure 2**. RT-PCR of RNA isolated from the cultures (steps 43-56 for stroma cells and 62-71 for organoid cultures) indicates the epithelial cellular marker *CDH1* is enriched in organoids (**Figure 2E**) and the stroma cell marker *HAND2* is enriched in stroma cells (**Figure 2F**). For the stromal cells, within 1-2 weeks colonies can be observed in the flasks. After 4-6 weeks, the first T25 flask of stroma cells will be confluent and can be passaged for expansion and cryopreservation. The passaged cells, seeded at 30-40% confluency, reach confluency within 1-2 weeks. We see variability in terms of organoid formation depending both on the amount of tissue in the menstrual fluid sample and individual donor variation in growth rates. From some samples, we obtain numerous organoids within 2-4 weeks, whereas few and more slowly growing organoids are formed from others. Often, initially, the organoid cultures will need to be replated/passaged despite the formation of only a few organoids due to the disintegration of Matrigel or to remove remaining non-epithelial cells (tissue debris, blood cells, and small presumed immune cells). One refinement to our method that would further enrich endometrial cells and decrease immune cells would be to employ the magnetic bead sorting described by Filby et al. (12) We have observed estrogen induction of *IHH* (**Figure 3A**), in organoid cultures (13), and indications of decidual response to estrogen, progesterone, and cAMP (EPC) treatment of stromal cells in terms of cell morphology (not shown) and induction of the decidual cell gene *PRL* (**Figure 3B**). Both of these responses are comparable to those observed in endometrial biopsy-derived cells. Other genes of interest that have been evaluated for estrogen response in organoids derived from endometrial biopsies include *GREB1* and *PGR* (13). Similarly, *IGFBP1* is another gene induced during decidualization (14).

**Table 4:** RT-PCR primer sequences.

CDH1-F	GTCATTGAGCCTGGCAATTTAG
CDH1-R	GTTGAGACTCCTCCATTCCTTC
HAND2-F	GCCAAGGACGACCAGAATGG
HAND2-R	GGTTTTCTTGTCGTTGCTGCT
PRL-F	GATGGAAGTCCCGACCAGAC
PRL-R	GGAGCAAACCAAACGGCTTC
IHH-F	GACCGCGACCGCAATAAGTA
IHH-R	TGGGCCTTTGACTCGTAATAC

All participants were between the ages of 18-35 years and had no known diagnoses of infertility, PCOS, or endometriosis. These menstrual fluid-derived cells exhibited inter-individual variability in hormonal responsiveness, as illustrated by the estrogen induction of *IHH* in organoids from three participants (**Figure 3A**) and the induction of the decidual marker *PRL* in stromal cells from the same three individuals (**Figure 3B**). This analysis illustrates that the relative responses between individuals differ in the two cell types. For example, participant A showed the most robust *PRL* induction in stromal cells (**Figure 3B**), whereas participant A’s *IHH* induction in organoids treated with estrogen was comparable to or less than that of the other two participants (**Figure 3A**).

## 5 Discussion

Model systems are crucial for experimental studies of human biology and disease. In the case of the human endometrium, well-established stromal culture systems that recapitulated hormone-dependent responses have been widely used (5). The culture of epithelial cells has proven more challenging, but the more recent development of organoid culture has led to an explosion of studies (7). Limitations of these systems include the wide variability in biological response characteristics between cultures derived from different individuals as well as the invasive procedure required to obtain endometrial samples for cell isolation. Utilizing menstrual fluid eliminates the need for endometrial biopsy, and greatly expands the breadth of potential donors, as the collection can be done without clinic visits. Ongoing studies will compare other characteristics, such as growth rates. Additionally, each participant can provide multiple samples, which will enable a longitudinal examination of the changes in the responsiveness of these tissues over time. Utilization of paired epithelial and stromal cultures from the same donor can be incorporated into studies as well.

The procedures employed to derive and maintain cultures are simple ones and can be done in any laboratory setting with access to basic mammalian cell culture equipment, supplies, and reagents. Therefore, we also think the procedure could be adapted for academic settings to serve the dual purpose of providing hands-on experience and training to life science undergraduate and graduate students while at the same time establishing cell lines with a broader representation of diverse communities. One limitation that could be a barrier for investigators is the costs of the reagents, especially for the Matrigel and for the additives that make up the organoid culture media. Recent work optimizing organoid culture media suggests the possibility of simplifying organoid ExM by omitting some of the additives or utilizing chemical inhibitors (15, 16). Similarly, the development of extracellular matrix hydrogels used in place of or in combination with Matrigel is not only less expensive but can also result in organoids that more closely resemble uterine tissue (17). Menstrual fluid-derived cultures are a promising readily available source of biological samples for *in vitro* experimental studies.

Uterine responsiveness requires contributions of the signals from cells in both stroma and epithelium and the interaction between these signals. Current work is being undertaken to establish coculture conditions of epithelium and stroma to be more reflective of the *in vivo* condition (18). This approach will allow both homologous and heterologous coculture of epithelium and stroma to determine the factors regulating endometrial responsiveness.

## Data availability statement

The original contributions presented in the study are included in the article/supplementary material. Further inquiries can be directed to the corresponding author.

## Ethics statement

The studies involving humans were approved by NIEHS Institutional Review Board. The studies were conducted in accordance with the local legislation and institutional requirements. Written informed consent was required and was obtained from participants themselves.

## Author contributions

SH, MD, KH, SG, and FD contributed to the conception and design of the study. SH and MD developed the method and conducted the experiments. NE and KH recruited the participants and coordinated sample collection and transport. SH and MD wrote the first draft of the manuscript. All authors contributed to the article and approved the submitted version.
